# Editorial: Genome Invading RNA Networks

**DOI:** 10.3389/fmicb.2018.00581

**Published:** 2018-03-27

**Authors:** Luis P. Villarreal, Guenther Witzany

**Affiliations:** ^1^Center for Virus Research, University of California, Irvine, Irvine, CA, United States; ^2^Telos–Philosophische Praxis, Buermoos, Austria

**Keywords:** RNA Networks, genetic identities, regulatory RNAs, infectious agents, Natural Genetic Content Operators

It has been long accepted that newly acquired biological information is mostly derived from random, error-based' events (Eigen, 1971). However, the serial nature of acquiring such random events makes it very difficult to account for the origin or modification of regulatory networks. There is now abundant empirical evidence establishing the crucial role of non-coding DNA (acting through the expression of RNA with its complex biology) to create regulatory control (Mattick, 2003; Atkins et al., 2011). Along with the parallel comeback of regulatory RNA in virology, RNA is now at center stage in how we think about complex organisms (Koonin et al., 2006; Atkins et al., 2011).

Regulatory RNAs derive from infectious events and can co-operate, build communities, generate nucleotide sequences de novo and insert/delete themselves into host genetic content (Villarreal, 2005; Koonin, 2009). In this sense genome invading RNA networks determine host genetic identities (self-recognition) throughout all kingdoms including the virosphere (Britten, 2004; Marraffini and Sontheimer, 2010; Villarreal, 2011a). But inclusion of a transmissive viral RNA biology differs fundamentally from conventional thinking in that it represents a vertical domain of life providing vast amounts of linked information not derived from direct ancestors (Villarreal, 2014). Interestingly single RNA stem loops react as physico-chemical entities exclusively, whereas with the network-cooperation of various RNA stem-loops in a module-like manner biological selection emerges (Manrubia and Briones, 2007; Vaidya, 2012; Higgs and Lehman, 2015). Additionally co-operating RNAs outcompete selfish genetic parasites (Hayden and Lehman, 2006; Vaidya et al., 2012).

Thus, we can argue, that for DNA based organisms, the introduction of infective collectives of RNA groups are a central driving force of evolution. Such RNA groups are co-adapted from persistent infectious agents and now serve as regulatory tools in nearly all cellular processes (Witzany, 2016) as documented in several retrovirus derived mobile genetic elements (Brosius, 1999; Villarreal, 2011b; Chuong et al., 2016). Additionally, the resulting productive RNA networks constantly produce new sequence space (i.e., complex regulation) which not only further serve as adaptation tools for their cell-based host organisms but also provides crucial roles in evolutionary novelty (Villarreal, 2011b). This RNA productivity results out of the empirical fact that a single RNA sequence can fold into different and unrelated secondary structures with different functions in a (environmentally determined) context-depending way (Schultes and Bartel, 2000).

Infection derived RNAs serve as the agents of regulatory networks in the cellular transcriptome (Feschotte, 2008; Briones et al., 2009; Koonin, 2009; Villarreal and Witzany, 2010). Without transcription from the genetic storage medium of DNA into the living world of such RNA agents, no relevant genetic process in the cellular transcriptome can be initiated (Volff, 2006). RNAs, with their inherent repeat syntax, format the expression of coding sequences and organize the coherent line-up of timely coordinated steps of replication (Shapiro and von Sternberg, 2005). The transport of genetic information to the progeny cells is also coordinated by these agents (Spadafora, 2017). Furthermore, they are crucial for the cooperation between networks of RNA-stem loops to constitute important nucleoprotein complexes such as ribosome, spliceosome, and editosome (Witzany, 2011). Therefore, such RNA groups are essential for complex order of genome constructions (Witzany, 2014).

Additionally of interest is that infectious non-coding RNAs insert preferentially in non-coding DNA areas, whereas coding DNA usually is not the target (Bushman, 2003; Mitchell et al., 2004; Bartel, 2009). In this perspective the non-coding DNA is the preferred habitat to settle down by infectious RNAs, e.g., y-chromosome in human genomes (Shapiro, 2002; Villarreal, 2009; Lambowitz and Zimmerly, 2011). This may indicate that the preferred change in evolutionary processes occurs in regulatory sections and not in the information storage coding for proteins, the main source for “mutations” in previous theoretical concepts of evolution (Villarreal and Witzany, 2013).

Frontiers Research Topic Genome Invading RNA Networks highlights various RNA networks being active in host genomes.

Sablok et al. discussed classification, identification and roles of tRNA derived smallRNAs across plants and their potential involvement in abiotic and biotic stresses. Wang et al. investigated how retrotransposon insertion polymorphisms can impact human health and disease. Moelling et al. demonstrated that RNase H-like activities of retroviruses, TEs, and phages, have built up innate and adaptive immune systems throughout all domains of life. Liu at al. summarize recent advances in understanding the roles of miRNAs involved in the plant defense against viruses and viral counter-defense. Malicki et al. review three retrotransposon classes that might represent a domestication of the selfish elements. Habibi and Salmani exemplified direct action of RNA networks in shaping the genome. Pecman et al. compared two different approaches for detection and discovery of plant viruses and viroids. Nagata et al. found that sequence changes in the RNase H domain and the reverse transcriptase connection domain are responsible for subtype classification. Zinad et al. suggest that natural antisense transcripts interfere with their corresponding sense transcript to elicit concordant and discordant regulation. Ottesen et al. describe how the abundance of Alu-like sequences may contribute toward Survival Motor Neuron gene pathogenesis. Ariza-Mateos and Gómez show how RNA viruses mimic key factors of the host cell. Spadafora found that spermatozoa act as collectors of somatic information and as delivering vectors to the next generation. Frías-Lasserre et al. demonstrate how current epigenetic advances on non-coding RNAs has changed the perspective on evolutionary relevant variations. Scolaro et al. demonstrate that evolutive processes for viruses are now interpreted as coordinated phenomenon that leads to global non-random remodeling of the population. Seligmann and Raoult found that ribosomal RNA stem-loop hairpins resemble those formed by viruses and short parasitic repeats infesting bacterial genomes. Fu et al. provide deep insights into the molecular mechanisms of influenza virus infection.

More and more empirical evidence establishes the crucial role of natural genetic content editors such as viruses and RNA networks to create genetic novelty, complex regulatory control, epigenetics, genetic identity, immunity, inheritance vectors, new sequence space, evolution of complex organisms and evolutionary transitions (Villarreal and Witzany, 2015; Chuong et al., 2016, Spadafora, this issue).

Genetic identities of RNA networks such as e.g., group I introns, group II introns, viroids, RNA viruses, retrotransposons, LTRs, non-LTRs, SINEs, LINEs, Alus invade and even persist in host genomes (Villarreal, 2009). Also mixed networks of RNA- and DNA viruses derived parts that integrate into host genomes have been found (Stedman, 2015), not forgetting persistent retroviral infections and the essential roles of reverse transcriptases and related RNase H endonucleases (Moelling and Broecker, 2015).

Highly dynamic RNA-Protein networks such as ribosome, editosome and spliceosome together with several context-dependent sequence modificating interactions, such as pseudo-knotting, frame-shifting, loop-kissing, by-passing translation generate a large variety of RNA regulatory functions out of a given DNA content (Cao et al., 2014; Denzler et al., 2014; Peselis and Serganov, 2014; Samatova et al., 2014; Keam and Hutvagner, 2015; Atkins et al., 2016).

There are reasonable expectations that this new empirically based perspective on the evolution of genetic novelty and biological information will have more explanatory power in the future than the “error-replication” narrative of the last century.

## Author contributions

All authors listed have made a substantial, direct and intellectual contribution to the work, and approved it for publication.

### Conflict of interest statement

The authors declare that the research was conducted in the absence of any commercial or financial relationships that could be construed as a potential conflict of interest.
